# The role of cAMP in synaptic homeostasis in response to environmental temperature challenges and hyperexcitability mutations

**DOI:** 10.3389/fncel.2015.00010

**Published:** 2015-02-02

**Authors:** Atsushi Ueda, Chun-Fang Wu

**Affiliations:** Department of Biology, University of IowaIowa City, IA, USA

**Keywords:** rutabaga adenylyl cyclase, quantal size, input resistance, synaptic growth, quantal content

## Abstract

Homeostasis is the ability of physiological systems to regain functional balance following environment or experimental insults and synaptic homeostasis has been demonstrated in various species following genetic or pharmacological disruptions. Among environmental challenges, homeostatic responses to temperature extremes are critical to animal survival under natural conditions. We previously reported that axon terminal arborization in *Drosophila* larval neuromuscular junctions (NMJs) is enhanced at elevated temperatures; however, the amplitude of excitatory junctional potentials (EJPs) remains unaltered despite the increase in synaptic bouton numbers. Here we determine the cellular basis of this homeostatic adjustment in larvae reared at high temperature (HT, 29°C). We found that synaptic current focally recorded from individual synaptic boutons was unaffected by rearing temperature (<15°C to >30°C). However, HT rearing decreased the quantal size (amplitude of spontaneous miniature EJPs, or mEJPs), which compensates for the increased number of synaptic releasing sites to retain a normal EJP size. The quantal size decrease is accounted for by a decrease in input resistance of the postsynaptic muscle fiber, indicating an increase in membrane area that matches the synaptic growth at HT. Interestingly, a mutation in *rutabaga* (*rut*) encoding adenylyl cyclase (AC) exhibited no obvious changes in quantal size or input resistance of postsynaptic muscle cells after HT rearing, suggesting an important role for *rut* AC in temperature-induced synaptic homeostasis in *Drosophila*. This extends our previous finding of *rut*-dependent synaptic homeostasis in hyperexcitable mutants, e.g., *slowpoke* (*slo*). In *slo* larvae, the lack of BK channel function is partially ameliorated by upregulation of presynaptic Shaker (Sh) IA current to limit excessive transmitter release in addition to postsynaptic glutamate receptor recomposition that reduces the quantal size.

## Introduction

Homeostatic mechanisms are involved in the striking ability of regaining stable synaptic efficacy or neural circuit performance following disturbances caused by environmental stressors or experimental insults in the nervous systems of a variety of species (Turrigiano and Nelson, 2004; Pérez-Otaño and Ehlers, 2005; Davis, 2006; Marder and Goaillard, 2006). In mammals, pharmacological manipulations of neuronal spiking or synaptic activities have been shown to cause compensatory changes in synaptic strength. For example, the blockade of action potentials by Tetrodotoxin (TTX) or postsynaptic inhibition by receptor antagonists can result in striking increases in spontaneous excitatory synaptic currents (EPSCs), as observed in dissociated cultures of cortical neurons (Turrigiano et al., 1998), hippocampal slices (Rao and Craig, 1997; Lissin et al., 1998), and *in vivo* recordings of visual cortical neurons (Desai et al., 2002) and spinal neurons (O’Brien et al., 1998). In some cases, the compensatory response is mediated through upregulation of receptor subtype expression (Watt et al., 2000; Leslie et al., 2001). However, the underlying general regulatory mechanisms and detailed molecular networks still await further elucidation.

A naturally occurring and ecologically relevant stressor that induces neuronal homeostatic adjustment is environmental temperature. Chronic temperature changes are known to affect neuronal development, leading to neuronal morphological alterations, such as neuronal dendritic field retraction during hibernation in ground squirrels (Popov and Bocharova, 1992; Popov et al., 1992; von der Ohe et al., 2006) or dendritic spine number reduction following short-term decreases in local temperature in the mouse brain (Kirov et al., 2004; Roelandse and Matus, 2004). Synaptic homeostatic adjustments also maintain the stability of physiological functions upon drastic temperature changes. For instance, in the stomatogastric ganglion of the crab phase relationships between action potential bursts characteristic to individual identified neurons are maintained despite temperature changes that significantly alter the neuronal firing rate within the central pattern generators (Tang et al., 2010, 2012). It is also known that after long-term exposure to extreme temperatures, neurons display adjustments in various physiological parameters, such as resting membrane potential and EPSP amplitude, in a number of species (fish: Roots and Prosser, 1962; Friedlander et al., 1976; snail: Merickel and Kater, 1974; crayfish: Harri and Florey, 1979; honeybee: Tautz et al., 2003; Groh et al., 2004; Jones et al., 2005).

The *Drosophila* larval neuromuscular junction (NMJ) offers abundant opportunities for studying the molecular and genetic mechanisms underlying synaptic homeostasis (Davis and Bezprozvanny, 2001 for review). It has been documented in *Drosophila* that axon terminal arborization is enhanced at increased temperatures (Sigrist et al., 2003; Zhong and Wu, 2004; Peng et al., 2007; Lee and Wu, 2010). These temperature-induced morphological alterations can be further modulated by mutations altering neuronal excitability: drastic increases by K^+^ channel mutations (Budnik et al., 1990; Zhong et al., 1992) and decreases by Ca^2+^ channel mutations (Lee and Wu, 2010). Significantly, such morphological modifications induced by increased temperature or neuronal hyperexcitability can be suppressed by a mutation in *rutabaga* (*rut*) encoding adenylyl cyclase (AC), implicating the involvement of the cAMP pathway in the plasticity of synaptic growth (Zhong et al., 1992; Lee and Wu, 2010).

Here we examine the physiological parameters of pre- and post-synaptic elements to identify the temperature-dependent alterations in maintaining. Such counterbalancing modifications enable the maintenance of stable synaptic transmission at the *Drosophila* larval NMJ upon high temperature (HT) rearing. In spite of the increased number of synaptic boutons, the excitatory junctional potential (EJP) size remains unaltered. Strikingly, this temperature-induced synaptic homeostasis was not observed in mutant larvae with impaired function of *rut* AC following HT rearing.

## Materials and methods

### *Drosophila* stocks

The *Drosophila melanogaster* stocks used include a wild-type (WT) strain Canton-S and a mutant *rutabaga^1^* (*rut^1^*). These lines have been previously described (Zhong et al., 1992; Kim and Wu, 1996; Renger et al., 2000; Peng et al., 2007). Flies carrying *UAS-rut^+^* was a generous gift from Dr. Troy Zars (University of Missouri, Columbia, MO, USA; Zars et al., 2000). A motor neuron-specific driver *C164-Gal4* (Torroja et al., 1999) and muscle-specific driver *mef2-Gal4* (Ranganayakulu et al., 1996) were used to drive expression of the transgene.

Fly stocks were maintained at room temperature (RT). However, the building RT varied significantly over the seasons between 1997 and 2001, as low as 15°C during winter and as high as 30°C during summer. Focal recording was carried out during this period. Experiments on rearing temperature effects and intracellular recording of EJP were performed after 2002 when the building temperature was maintained at 22–24°C throughout the year. To examine the effect of rearing temperature, we compared the stocks maintained at RT with those reared in 29–30°C incubators.

### Larval neuromuscular preparations and physiological solutions

**Figure 1:**
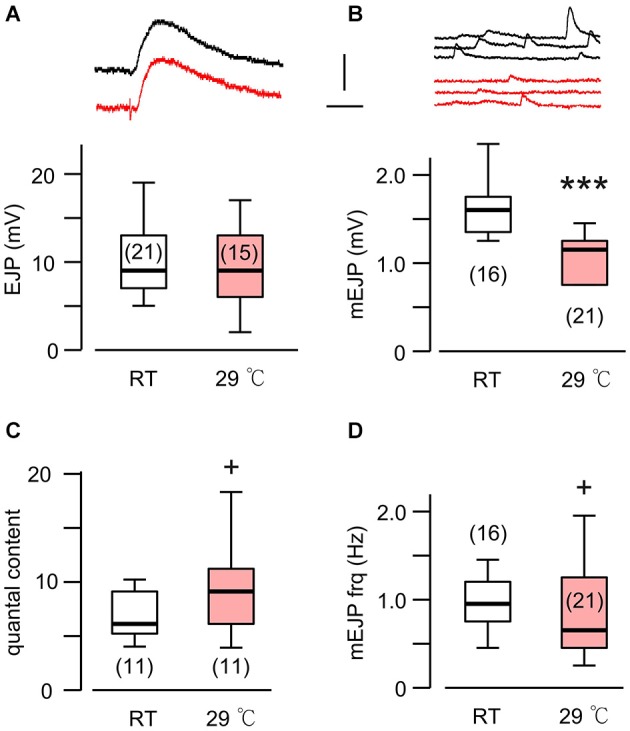
**A novel form of synaptic homeostasis in response to high temperature rearing**. **(A)** Evoked EJP size was not affected by rearing temperatures. Upper (black) and lower (red) traces are from WT larvae reared at room temperature (23°C, RT) and high temperature (29°C, HT), respectively. **(B)** Miniature EJPs (mEJPs) became smaller when reared at HT. ***p < 0.001, *t*-test. **(C)** Quantal content (ratio of mean amplitudes of EJP to mEJP) in HT-reared larvae could be larger than that in RT-reared larvae, as indicated by a skewed distribution. + *p* < 0.05, *F*-test. **(D)** Frequency of mEJPs in HT-reared larvae was also more variable than that in RT-reared larvae. + *p* < 0.05, *F*-test. Scale bars: 5 mV and 20 ms **(A)** or 200 ms **(B)**. Number of muscle fibers indicated in parenthesis. Data recorded in HL3.1 saline containing 0.2 mM **(A,C)** or 0.2 and 0.5 mM Ca^2+^ **(B,D)**. **(A)** is constructed from data in Ueda and Wu (2012) with additional data.

**Figure 2:**
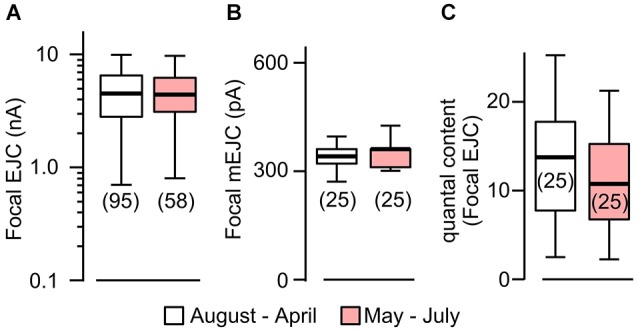
**Focal mEJCs and EJCs were not affected by rearing temperature**. Focal EJCs **(A)** and mEJCs **(B)** remained stable despite ambient temperature fluctuations. Building temperature varied significantly: from as low as 15°C to beyond 30°C (mostly during May to July). Pooled data from August to April are compared to those from May to July. **(C)** Quantal content of the focal EJCs was not different between the two periods (*p* > 0.5, Wilcoxon rank-sum test). Number of recording sites indicated in parenthesis. **(A)** is reconstructed from data in Ueda and Wu (2012).

**Figure 3:**
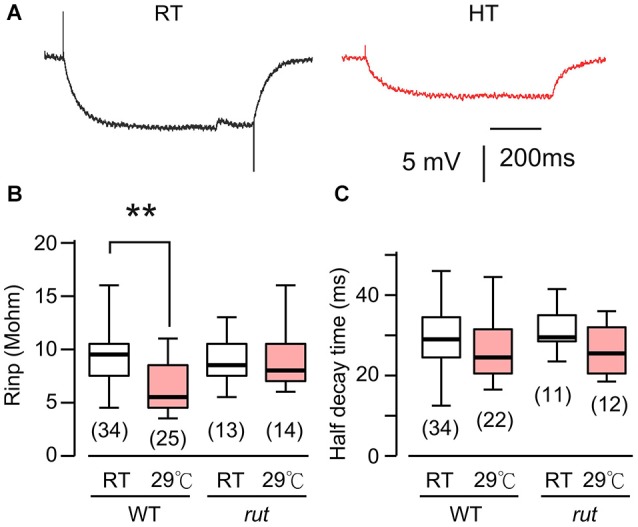
**HT rearing decreased muscle input resistance in WT, but not *rut* larvae. (A)** Muscle membrane potential traces in response to hyperpolarizing current injection in WT larvae reared at RT and HT. **(B)** Input resistance was decreased in HT-reared WT, but not *rut*, larvae. **(C)** Rearing temperature did not significantly affect half-decay time in WT and *rut*. *p < 0.05, One-way ANOVA. Number of muscle fibers indicated in parenthesis. Data from 0.2 and 0.5 mM Ca^2+^ were pooled. HL3.1 saline.

**Figure 4:**
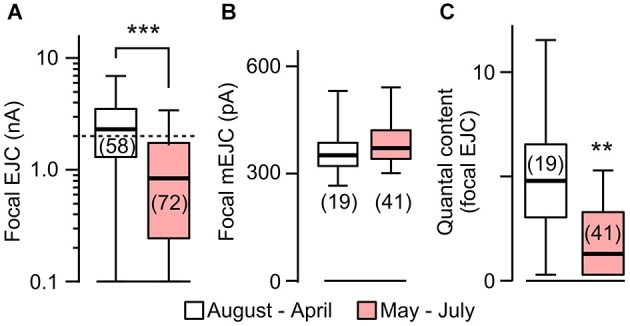
**Focal EJC amplitudes affected by rearing temperature in *rut*. (A)** Focal EJCs from the great majority of *rut* boutons were below 2 nA (dotted line) during the period of May to July, whereas those collected outside of this period were substantially larger. Compare to Figure 2 for constant EJC size for WT. **(B)** Focal mEJC amplitudes in *rut* did not vary with ambient temperature fluctuations. **(C)** Quantal content was significantly smaller at HT. Number of recording sites indicated in parenthesis. ***p < 0.001; **p < 0.01, Wilcoxon rank-sum test. **(A)** is reconstructed from data in Ueda and Wu (2012).

**Figure 5:**
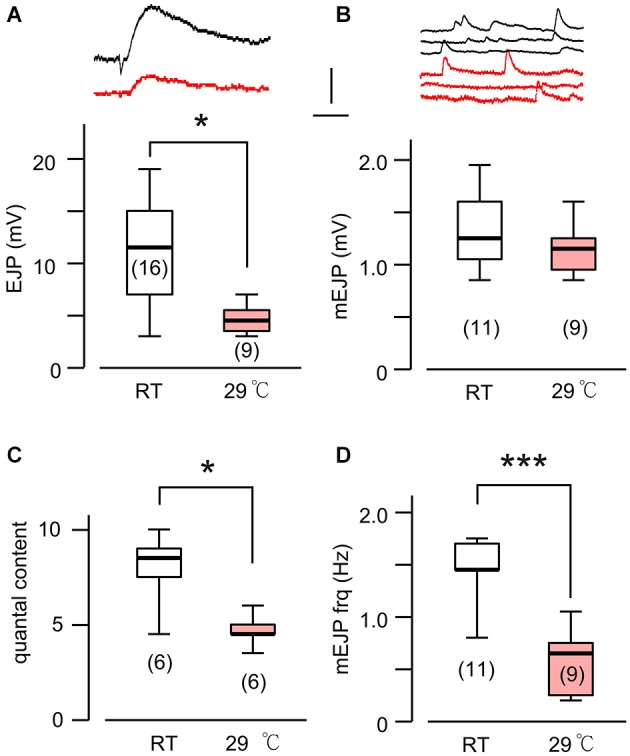
**Altered transmitter release in HT-reared *rut* larvae**. **(A)** Evoked EJP size became drastically smaller in *rut* when reared at HT. Upper (black) and lower (red) traces are from RT- and HT- reared larvae, respectively. In *rut*, mEJP size was not significantly altered by HT rearing **(B)**, although quantal content was significantly reduced **(C)**. Frequency of mEJP was reduced in *rut* when reared at HT **(D)**. ***p < 0.001; *p < 0.05, *t*-test. Number of muscle fibers indicated in parenthesis. Data recorded in HL3.1 saline containing 0.2 mM **(A,C)** or 0.2 and 0.5 mM Ca^2+^ **(B,D)**. **(A)** is constructed from data in Ueda and Wu (2012) with additional data.

**Figure 6:**
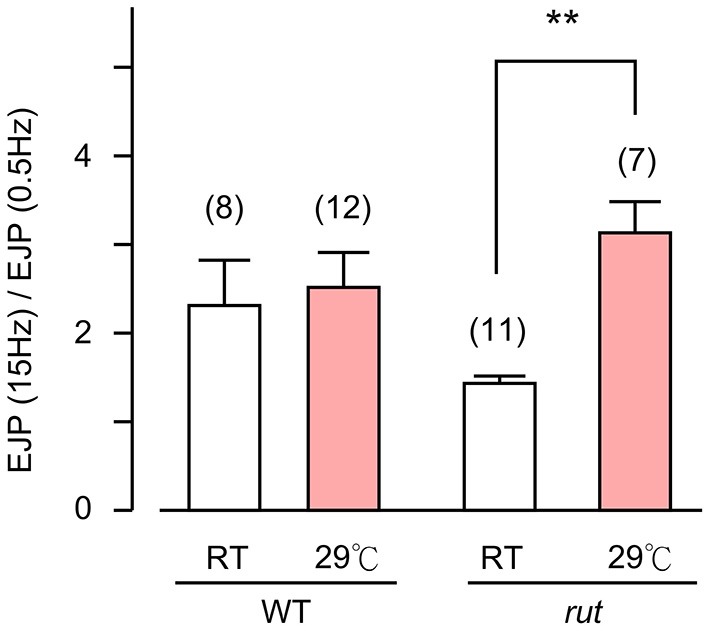
**Altered activity-dependent facilitation of EJPs in *rut* larvae**. EJPs in *rut* larvae displayed enhanced facilitation when reared at HT. Ratios of EJP amplitudes evoked by 15-Hz–0.5 Hz provides an index for facilitation. Stimulus trains (10 s, 15 Hz) were applied and average EJP amplitudes were determined between 5–10 s. **p < 0.01, *t*-test with sequential Bonferroni adjustment for multiple comparisons of RT vs. HT. Number of muscle fibers indicated in parentheses. Error bars indicate SEM.

Post-feeding third instar larvae were dissected in Ca^2+^ free HL3 saline (Stewart et al., 1994) containing (in mM) 70 NaCl, 5 KCl, 20 MgCl_2_, 10 NaHCO_3_, 5 Trehalose, 115 Sucrose, and 5 HEPES, at pH 7.2. For physiological recordings, we used either HL3 (focal recording in Figures 2, 4) or HL3.1 (Feng et al., 2004), which have the same ionic composition except for a reduced Mg^2+^ concentration in HL3.1 (4 mM, whole cell intracellular recordings, Figures 1, 3, 5, 6). The final Ca^2+^ concentration in recording saline is specified for each experiment. To evoke nerve action potentials and excitatory junctional currents (EJCs), the segmental nerves were severed from the ventral ganglion and stimulated with a suction electrode (10 µm inner diameter) through the cut end. Stimulation amplitude was adjusted to 2.0–2.5 times the threshold voltage to ensure a uniform stimulation condition among experiments. Stimulus duration was 0.1 or 0.5 ms.

### Focal loose patch-clamp recording

Extracellular focal recordings were performed as described previously (Renger et al., 2000; Ueda and Wu, 2009). Briefly, fire-polished focal recording electrodes with an inner diameter of 4–8 µm and an outer diameter of 15–20 µm were filled with HL3 saline and were placed over type I boutons on muscle 13. The pipette opening typically covered one type Ib bouton. EJC signals were picked up with a loose-patch clamp amplifier (Patch Clamp 8510; Zeitz Instruments, Munich, Germany) and stored on VCR tapes with a Pulse Code Modulator (Neuro Data, model Neuro-Corder DR-384, New York, NY). All trials contained a calibration pulse to determine the electrode series and seal resistance in order to correct for current leakage at the pipette tip (Stühmer et al., 1983). In rare occasions, biphasic currents were observed, but they were excluded from data analysis.

### Whole-cell excitatory junctional potential (EJP) and muscle input resistance measurements

Nerve-evoked neurotransmitter release was also recorded intracellularly from postsynaptic muscle fiber 6. Intracellular glass microelectrodes were filled with 3 M KCl and had a series resistance of about 60 MΩ. EJPs were picked up with a direct current pre-amplifier (model M701 micro-probe system, WPI, Conn., USA, and an additional custom-built amplifier). Muscle membrane resistance was measured by injecting −1 nA current pulses with 700 ms duration into a muscle cell. A bridge circuit was used to measure the membrane potential change. We selected cells with resting potentials deeper than −55 mV for evoked EJPs and muscle input resistance measurement. For miniature EJP (mEJP) measurement, we selected cells with resting potentials deeper than −60 mV to ensure minimal membrane damage caused by electrode penetrations.

### Statistical analysis

As described in the Results, ANOVA, *F*-test, Wilcoxon rank-sum test, and student *t*-test were carried out with sequential Bonferroni adjustment for multiple comparisons.

## Results

### Homeostasis of synaptic efficacy upon HT rearing: Maintained EJP amplitude with increased bouton numbers and reduced mEJP size

It is now well established that HT rearing (at 29°C) induces synaptic terminal overgrowth resulting in an increased number of presynaptic boutons at the *Drosophila* larval NMJ (Sigrist et al., 2003; Zhong and Wu, 2004; Lee and Wu, 2010). However, the synaptic strength, as determined by the amplitude of the EJPs in the postsynaptic muscle cell, remains unaltered compared to that in larvae reared at RT (Figure 1A; Ueda and Wu, 2012). This raises the question of how the number of presynaptic vesicles released from individual boutons and the postsynaptic response to individual vesicles are adjusted in order to maintain stable synaptic efficacy when reared at HT. To answer this question, we first carried out intracellular recording to measure the muscle whole-cell response (EJP) and the spontaneous miniature EJPs (mEJPs), which reflect the postsynaptic response to spontaneous release of single vesicles (quantal size, Figure 1B). These parameters allowed us to estimate the quantal content, i.e., the number of vesicles released, underlying each EJP.

We used saline containing low Ca^2+^ (0.2 mM), at which the summation of mEJPs is nearly linear, circumventing the problem of nonlinear summation, allowing for the simple determination of quantal contents by the index [EJP]/[mEJP], where [EJP] and [mEJP] represent the average amplitude of EJPs and mEJPs. Our data collected from muscles 6 and 7 indicate that the quantal size, as determined from the mEJP size, was reduced in WT when reared at HT (Figure 1B). To maintain the same EJP size, this led to an increase in quantal content (Figure 1C). Interestingly, a previous study also reported a similar percentage increase in the number of boutons in muscles 6 and 7 after HT rearing at 29°C (Sigrist et al., 2003). Therefore, it is important to investigate how HT-rearing affects the functioning at the synaptic bouton level, in terms of the number of vesicles released upon nerve stimulation and the postsynaptic currents generated by each vesicle in order to account for the ensemble EJP response evoked by each nerve action potential.

### Rearing-temperature effect on postsynaptic currents generated by individual boutons

We have previously established focal loose-patch recordings to determine local synaptic currents generated from transmitter release within individual boutons (Kurdyak et al., 1994; Renger et al., 2000; Ueda and Wu, 2009, 2012). Using a focal pipet electrode of a standard configuration and size (see Section Materials and Methods), local EJCs, as well as spontaneous miniature EJCs (mEJCs), from boutons under the patch electrode can be determined to complement whole-cell intracellular recordings of spontaneous mEJPs and evoked EJPs.

To quantify temperature-induced changes in focal mEJCs and EJCs, we performed loose-patch clamping followed by the established procedure to correct for errors introduced by leakage currents (see Section Materials and Methods; Kurdyak et al., 1994). We took advantage of a large data set collected between 1997–2000, during which ambient temperature for fly rearing and physiological recording varied between different seasons, approaching 30°C in the summers and 15°C in the winters (see Section Materials and Methods; Ueda and Wu, 2012). We followed chronologically the data throughout these years and found no variation in EJC amplitude despite the temperature variation (Figure 2A; Ueda and Wu, 2012). When we performed additional analysis to include mEJC size using the same set of focal recording data, we found that focal mEJC size was also unaltered (Figure 2B). The fact that focal mEJCs remained the same is consistent with the simple interpretation that rearing temperature affects neither the amount of transmitter released per synaptic vesicle, nor the postsynaptic glutamate receptor channel response to each vesicle release. Therefore, unaltered focal EJCs and mEJCs support the conclusion that the quantal content for the local release from each bouton was unaffected by temperature variations (Figure 2C). Overall, this is consistent with the notion that the increased whole-cell quantal content determined by EJP measurements (Figure 1C) simply reflects the increase in presynaptic bouton numbers.

It should be noted that focal mEJCs amplitude did not appear to vary at different Ca^2+^ levels. Although the focal mEJC analyses in Figure 2 were performed at a physiological Ca^2+^ level (1.5 mM), the results were compatible with the whole-cell mEJP recording that were collected at a low Ca^2+^ concentration (0.2 mM, Figure 1 to avoid nonlinear summation distortion, see Section Materials and Methods). Additional focal mEJC measurements performed at a lower Ca^2+^ concentration (0.5 mM) produced similar results (330 ± 67 pA at 0.5 mM, *n* = 10 fibers vs. 340 ± 45 pA at 1.5 mM, *n* = 50 fibers).

### Decreased postsynaptic muscle input resistance accounts for mEJP size decrease in HT-reared larvae

The postsynaptic voltage responses, mEJPs and EJPs, reflect charging of muscle membrane capacitance by the synaptic currents, mEJCs and EJCs. The disparity between the HT-rearing effects on mEJP size and mEJC size prompted us to examine the passive electrical properties of the postsynaptic muscle fiber (i.e., muscle cell membrane resistance and time constant). To determine these parameters, we measured the voltage response to hyperpolarizing negative current injection of fixed amplitudes (−1 to −2 nA). The results showed a significant decrease in muscle input resistance in HT-reared larvae (Figures 3A,B). This indicates that when focal mEJCs remain unaltered, an increase in membrane conductance may be responsible for the diminished mEJPs in HT-reared larvae (Figure 1B). Indeed, we found a positive correlation between input resistance and mEJP size among individual muscle fibers (correlation coefficient = 0.59, *p* < 0.01, data not shown). Furthermore, there was no clear indication of changes in the membrane time constants as evidenced by unaltered half decay time upon cessation of hyperpolarizing current injection (Figure 3C). The results suggest no changes in the passive muscle membrane properties (membrane resistance and capacitance per unit area).

Taken together, for the postsynaptic muscle fiber as a functional unit, the whole-cell EJP size is homeostatically maintained after HT rearing. In spite of the increase in synaptic bouton numbers leading to the increase in quantal content ([EJP]/[mEJP]), stable synaptic strength is maintained through the counterbalancing effect of the decrease in postsynaptic muscle input resistance.

### A role of rut AC in synaptic homeostasis: diminished HT-rearing effects on muscle input resistance and quantal events

Previous *Drosophila* studies have established a clear role of cAMP in synaptic overgrowth induced by HT rearing or hyperexcitability mutations because mutations in *rut* AC suppress NMJ arborization or neuronal branching under such conditions (Zhong and Wu, 2004; Peng et al., 2007; Lee and Wu, 2010). Therefore we first examined the muscle input resistance of *rut* mutant larvae to determine whether the cAMP pathway is also involved in the HT-induced decrease in muscle input resistance. We observed a clearly different response to HT rearing in *rut* compared to WT larvae. When comparing RT-reared and HT-reared *rut* larvae, the muscle input resistance and membrane time constant remained unaltered, as evidenced by the similar responses to the same hyperpolarizing current injection (Figures 3B,C).

The above results prompted us to compare the sizes of mEJCs in RT- and HT-reared *rut* larvae (Figure 4) using loose-patch focal recording. We analyzed the same sets of focal recording data from 1997–2000 (Ueda and Wu, 2012) in parallel with the analysis for WT larvae (Figure 2). The results showed that the quantal unit of transmission, focal mEJCs (Figure 4B), was not altered by HT rearing in *rut* larvae, similar to the observation in WT larvae. However, synaptic efficacy in *rut* is drastic reduced by HT rearing, as reflected by diminished focal EJCs (Figure 4A; Ueda and Wu, 2012).

Based on the fact that the bouton number in *rut* larvae remains unchanged following HT rearing (Zhong and Wu, 2004), one should be able to predict the properties of EJPs in *rut* larvae following HT rearing. As a functional unit, the *rut* muscle fiber would display an unaltered size of quanta (mEJPs) but greatly diminished EJPs coupled with a reduction in quantal content. Our intracellular recording confirmed these predictions. Although the *rut* EJP amplitude was not significantly different from WT larvae when reared at RT, *rut* EJPs drastically reduced in size following HT rearing, in striking contrast to the homeostatic maintenance of EJPs in WT (Compare Figures 1A and 5A). Furthermore, following HT rearing the mEJP size remained the same in *rut*, unlike a reduction in WT (Compare Figures 1B and 5B). Correspondingly, HT rearing exerted opposite effects on the quantal content in *rut* (decrease) vs. WT (increase) (Compare Figures 1C and 5C).

Finally, an examination of the spontaneous mEJP frequency revealed another transmitter release defect caused by *rut* upon temperature challenges. While HT rearing did not change the mEJP frequency in WT, there was a drastic decrease in spontaneous mEJP frequency in *rut* (Compare Figures 1D and 5D). More extensive temperature-dependent defects in *rut* were also evident in short-term activity-dependent plasticity following HT rearing. Using a synaptic augmentation protocol (Zhong and Wu, 1991; Renger et al., 2000) with high-frequency nerve stimulation (15 Hz, 10 s, 0.2 mM Ca^2+^), *rut* larvae displayed drastically increased augmentation following HT rearing, whereas WT larvae retained the same augmentation properties. Therefore, short-term synaptic plasticity is also maintained in HT-reared WT larvae but disrupted in *rut* larvae with a striking enhancement of activity-dependent synaptic facilitation following HT rearing (Figure 6).

Our previous study has manipulated the expression of *rut* AC separately in pre- and post-synaptic compartments to examine the consequences on *rut* morphological phenotypes. With targeted expression of *UAS-rut^+^* by neuron- or muscle-specific *Gal4* drivers, it has been shown that the HT-induced NMJ overgrowth can be rescued by neuronal, but not muscular, expression of *rut^+^* (Zhong and Wu, 2004). Here we asked how pre- or post-synaptic expression of *rut^+^* modifies the defective synaptic homeostasis in HT-reared *rut^1^* larvae. To express *rut^+^* in motor neurons and muscles in *rut^1^* mutant background, we drove *UAS-rut^+^* expression by *C164-Gal4* (Torroja et al., 1999) and *mef2-Gal4* drivers (Ranganayakulu et al., 1996), respectively. We found that neither driver could totally rescue the *rut^1^* phenotypes. Nevertheless, overexpression of *rut^+^* in muscle restored one aspect of the HT-induced homeostasis, i.e., EJP amplitude adjustment, but it did not modify muscle input resistance to mimic the WT response. In contrast, presynaptic overexpression of *rut^+^* in motor neurons did not rescue either of the HT-induced homeostasis.

For EJP amplitudes, muscle expression of *rut^+^* appeared to rescue the homeostatic maintenance (15.4 ± 4.2 and 16.6 ± 4.7 mV, *n* = 8 and 7 fibers, respectively, for RT and HT rearing, *p* > 0.05 with *t*-test), whereas expression of *rut^+^* in motor neurons lead to no restoration (21.6 ± 6.0 and 12.9 ± 7.7 mV, *n* = 7 and 7, *p* < 0.05). For the case of muscle input resistance, neither neuronal or muscle expression of *rut^+^* could rescue the expected HT-induced decrease, as seen in WT (neuronal expression: 7.8 ± 3.3 and 8.5 ± 2.5 Mohm, *n* = 13 and 15, *p* > 0.05; muscle expression: 7.3 ± 1.9 and 6.5 ± 2.0 Mohm, *n* = 15 and 15, *p* > 0.05).

## Discussion

### Synaptic homeostasis at the *Drosophila* NMJ

The *Drosophila* larval NMJ has been widely used for studying the genetic and molecular mechanisms underlying synaptic transmission and plasticity. Our study highlights a distinct category of synaptic homeostasis in response to environmental temperature stress. Our finding also indicates the involvement of the cAMP pathway in this form of homeostatic adjustment, further illustrating the important role of cAMP previously implicated in the various forms of synaptic and behavioral plasticity in *Drosophila* (Engel and Wu, 2009; Bushey and Cirelli, 2011; Kahsai and Zars, 2011; Ueda and Wu, 2012).

The first documented case of synaptic homeostasis at the *Drosophila* larval NMJ originates from a reduction in synaptic bouton numbers in mutants of the cell adhesion molecule Fasculin II (Stewart et al., 1996). This severe developmental alteration of the NMJ size nevertheless does not change the EJP amplitude, due to a compensatory increase in neurotransmitter release from individual synaptic boutons. Subsequent studies based on manipulations to decrease the postsynaptic quantal response further demonstrate the robust homeostatic adjustment in transmitter release at larval NMJs. Compensatory increases in the number of synaptic vesicle release (i.e., quantal content) occurs when the mEJP amplitude (quantal size) is diminished following either mutational disruption (Petersen et al., 1997; DiAntonio et al., 1999) or pharmacological blockade (Frank et al., 2006) of the postsynaptic glutamate receptors. In addition, when the mEJP amplitude is diminished due to a decrease in muscle input resistance by forced expression of inward rectifier K^+^ channels (Kir; Baines et al., 2001), synaptic vesicle release from individual boutons is also homeostatically upregulated to maintain a stable EJP amplitude (Paradis et al., 2001).

A mechanism of retrograde trans-synaptic signaling via yet to be identified postsynaptic factors has been implicated in further studies. However, these studies also indicate that bone morphogenesis protein (BMP), encoded by *glassbottom boat* (*gbb*), is required for synaptic homeostasis (Goold and Davis, 2007; Frank et al., 2009). The action of BMP during the early phases of embryonic and larval development enables the HT- and hyperexcitability-induced synaptic growth that occurs at later stages of larval development (Berke et al., 2013). In addition, a number of presynaptic proteins also participate in the regulation of synaptic homeostasis, including Ephexin, a Rho-type guanine nucleotide exchange factor (Frank et al., 2009), *cacophony-*encoded voltage-gated calcium channels, (Frank et al., 2009; Müller and Davis, 2012; Lee et al., 2013), and a GTPase-activating protein Rab3 (Müller et al., 2011).

### Temperature-dependent homeostatic adjustments of physiological parameters in maintaining stable synaptic transmission

Distinct from the above examples of synaptic homeostasis, our study reports a different category of synaptic plasticity upon long-term exposure to an environmental stressor, i.e., HT. This reflects an intrinsic ability of neuromuscular adaptation to ensure animal survival at stressful temperatures. Although the HT-induced increase in synaptic bouton numbers results in larger whole-cell synaptic currents (EJCs) (Sigrist et al., 2003), the EJP size recorded intracellularly in the postsynaptic muscle retains a level similar to that in RT-reared larvae (Ueda and Wu, 2012; Figure 1). Our data provide an explanation: A larger synaptic current does not lead to an increase in synaptic potential owing to a corresponding decrease in muscle input resistance.

Our results show that the postsynaptic current generated by each bouton actually remains unaltered following chronic HT exposure, as evidenced by constant sizes of the focal EJC and mEJC, indicating no changes in quantal content for the release from individual boutons (Figure 2C). The mEJP amplitude is nevertheless reduced due to a lower input resistance of the muscle membrane (Figure 3). Therefore, upon arrival of each action potential, the ensemble number of transmitter vesicles released from the axonal terminal is increased because of a larger number of NMJ boutons, but this does not cause a larger postsynaptic muscle EJP due to a counterbalancing decrease in muscle input resistance. The simplest explanation is that long-term HT exposure induces the coordinated growth of both presynaptic nerve terminals and post-synaptic muscle such that a stable synaptic efficacy or muscle response is maintained. At present time, it is unclear whether the muscle growth precedes the nerve terminal ramification or the other way round during the process of HT-induced homeostatic adjustment, a topic that awaits further investigation.

These findings are largely consistent with previously publications (Sigrist et al., 2003; Berke et al., 2013), which reported NMJ overgrowth and increased EJC quantal content following HT treatment. However, it should be noted that in the voltage-clamp study of EJCs, Sigrist et al. (2003) did not report a decrease in the muscle input resistance of HT-reared larvae, i.e., the input resistance is about 6 Mohm, independent of rearing temperature. In contrast, our measurements based on current injection were 9.6 and 6.6 Mohm for RT- and HT-reared WT larvae, respectively (Figure 3). The reason for this difference is unknown. However, we note that the input resistance and mEJP amplitude for individual muscle fibers are highly variable, as evidenced from the ranges of data reported here (Figures 1, 3). Thus, we base our conclusions on measurements from large samples of muscle fibers that met a stringent resting potential criterion to exclude muscles with membrane damage (see Section Materials and Methods).

Decreases in larval muscle input resistance have also been observed following acute heat treatments (Barclay and Robertson, 2003) or high-frequency nerve stimulation (Gertner et al., 2014), again highlighting the parallel between the effects of HT and hyperexcitability (Sigrist et al., 2003; Zhong and Wu, 2004). However, in these cases the input resistance decrease can be observed within a relatively short time period of a few tens of minutes, distinct from our long-term rearing effect following days of HT exposure. Acute heat-treatment results in decrease in both muscle input resistance and time constant (Barclay and Robertson, 2003), indicating no drastic changes in the total membrane capacitance (proportional to membrane area; Hille, 2001) of the muscle fiber. In contrast, our study does not indicate a HT rearing-induced change in membrane time constant (Figure 3C), reflecting an increase in effective membrane area without altering passive membrane properties (Hille, 2001) of the muscle fiber.

Interestingly, high-frequency (20 Hz) nerve activity is equally effective in decreasing larval muscle cell input resistance within a few tens of minutes (Gertner et al., 2014). Notably, this stimulus frequency is comparable to motor neuron firing rates during fictive locomotor activity in dissected larval preparations (Budnik et al., 1990; Fox et al., 2006; Chouhan et al., 2010). Also, the decline in the input resistance involves Ca^2+^-dependent mechanisms since it is suppressed by genetic or pharmacological perturbations in a Ca^2+^-activated K^+^ (SK) channels or Ca^2+^-dependent protein phosphatase 2A (Gertner et al., 2014). Interestingly, the *rut^1^* mutation used in our study also abolishes the Ca^2+^-dependence of AC activity (Dudai and Zvi, 1984; Livingstone et al., 1984).

### Rut AC in synaptic homeostasis and neural plasticity at different time scales

We found that decreases in muscle input resistance following HT rearing were not observed in *rut* mutant larvae defective in AC, suggesting a role of cAMP in the homeostatic regulation mechanisms. In parallel, previous reports also document that *rut* mutations suppress HT-induced enhancement in neuronal growth, including dissociated neurons in culture (Peng et al., 2007), larval NMJ arbors (Zhong and Wu, 2004; Lee and Wu, 2010), and adult mushroom body neurons (Peng et al., 2007). These findings demonstrate the importance of cAMP signaling in the long-term adjustment of neuronal growth and function.

It should be noted that *rut* AC appears to be pervasively involved in different forms of activity-dependent neural plasticity, spanning a wide range of time scales, from seconds and minutes to hours and days. Within the range of seconds, activity-dependent, short-term plasticity of synaptic efficacy is well known to be altered by mutations of *rut* and* dunce* (*dnc*, encoding cAMP-specific phosphodiesterase), such as synaptic facilitation or depression at larval NMJs (Zhong and Wu, 1991; Renger et al., 2000; Ueda and Wu, 2009). Imaging studies of dissociated neurons in both *dnc* and *rut* cultures have also demonstrated altered growth cone motility (Kim and Wu, 1996) and abnormal Ca^2+^ transients in different neuronal compartments in the time frame of seconds (Berke and Wu, 2002). At more extended time scales of minutes, *dnc* and *rut* mutations affect activity-dependent recruitment of synaptic vesicles from the reserve pool (Kuromi and Kidokoro, 2000) and post-tetanic potentiation of transmitter release (Zhong and Wu, 1991) at the larval NMJ, as well as the habituation process of the jump-and-flight escape reflex mediated by the adult giant fiber (Engel and Wu, 1996).

With its wide ranging effects on activity-dependent neuronal plasticity, it is not surprising that the *rut* NMJs display detectable alterations in basic transmission properties. In addition to modified synaptic facilitation mentioned above (see also Figure 6), *rut* NMJs are known to have a range of alterations even at RT. For example, Ca^2+^ imaging demonstrates that influx through Ca^2+^ channels is more sensitive to Co^2+^ blockade; focal loose-patch recording reveals that vesicular release of transmitter appears to lack synchrony; and ultrastructural studies demonstrate a reduced number of docked vesicles and an increased area of synaptic density (Renger et al., 2000; Ueda and Wu, 2009). In the present study, we observed a tendency of increased frequency of spontaneous mEJP (compare Figures 1D and 5D) and decreased augmentation (Figure 6) in *rut* larvae reared at RT. HT rearing further modifies some of these altered properties, including a reduction in mEJP frequency and a decrease in release efficacy, i.e., reduced quantal content (Figure 5).

For different forms of neuronal plasticity, the role of *rut* AC may vary in terms of the exact molecular mechanisms and cellular compartments of its action. As reported above, the Gal4-UAS experiments raise the possibility of *rut* AC involvement in pre- and post-synaptic interactions for the HT-induced synaptic homeostasis. We present below a comparison of hyperexcitability- and HT-induced synaptic homeostatic regulations that involve a diversity of molecular players and cellular mechanisms orchestrated by cAMP signaling in the pre- and post-synaptic compartments.

### Hyperexcitability- and HT-induced homeostatic regulation of synaptic function during development

On the developmental time scale of hours to days, cAMP plays an important role in HT rearing- or hyperexcitability-induced overgrowth of nerve terminals. As described above, HT-rearing increases synaptic bouton numbers in WT larvae, which is suppressed by *rut* mutations (Zhong and Wu, 2004). Similarly, hyperexcitability mutations of various K^+^ channels are known to promote nerve terminal overgrowth at the NMJ (reviewed in Fox et al., 2005). These K^+^ channels include Eag or Kv10 encoded *by ether a-go-go* (*eag*, Warmke et al., 1991), Shaker or Kv1 encoded by *Shaker* (*Sh*, Kamb et al., 1987; Papazian et al., 1987; Pongs et al., 1988), Slo BK encoded by *slowpoke* (*slo*, Atkinson et al., 1991), and Erg or Kv11 encoded by *seizure* (*sei*, Titus et al., 1997; Wang et al., 1997). In many of these cases, the mutational effects on NMJ synaptic overgrowth are further enhanced by increased cAMP levels in *dnc* mutant backgrounds but suppressed by defective AC activity in *rut* mutant backgrounds (Zhong et al., 1992; Lee and Wu, 2010).

Importantly, HT rearing also further enhances the potency of NMJ overgrowth in several of these K^+^ channel mutations, including *Sh* (Zhong and Wu, 2004) as well as *slo* and *sei* (Lee and Wu, 2010). The same is true for HT treatment on hyperexcitability-induced neurite overgrowth in the adult mushroom body, as well as cultured neurons of *eag* and *Sh* mutants (Peng et al., 2007). Our findings of the HT rearing-induced decrease in muscle input resistance accompanying synaptic bouton growth adds another dimension of the *rut* AC action on homeostatic functional matching between the pre- and post-synaptic elements.

**Figure 7:**
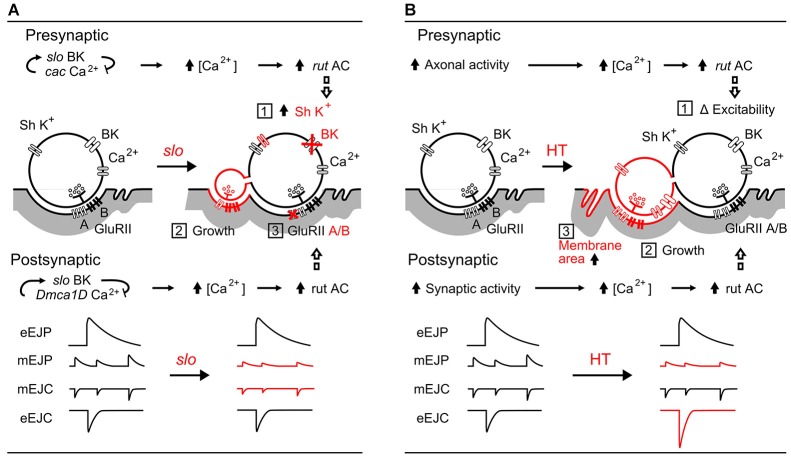
**Comparisons between *slo* hyperexcitabilty mutation- and HT rearing-induced synaptic homeostasis. (A)** *slo* mutation-induced pre- and post-synaptic homeostatic adjustment (Lee et al., 2008, 2013; Lee and Wu, 2010): (1) upregulation of presynaptic *Sh* I_A_ K^+^ currents to compensate for the loss of *slo* BK currents, (2) growth of excessive, functional satellite boutons, and (3) altered GluRII receptor composition (subunit A vs. B ratio) resulting in decreased mEJP amplitude. The roles of *cac* and *Dmca1D* Ca^2+^ channels, as well as the *rut* AC-mediated adjustments are depicted. **(B)** HT rearing induced-homeostasis: (1) regulation of excitability by *rut* AC (Ueda and Wu, 2009), (2) increased number of boutons (Zhong and Wu, 2004; Berke et al., 2013), and (3) decreased muscle membrane resistance from increased effective muscle membrane area. The resultant modifications in amplitudes of mEJPs and EJPs are summarized. In both **(A)** and **(B)**, *rut* AC plays an important role in homeostatic regulation of both synaptic function and growth.

The apparent parallel between the effects of hyperexcitability and HT rearing may be deceptive in terms of their underlying molecular and cellular mechanisms, even though *rut* AC plays important role in both cases. The hyperexcitable *slo* mutants have been studied in great detail for their pre- and post-synaptic readjustments to attain synaptic stability (Lee et al., 2008). Presynaptic terminals in *slo* mutants lack the critical regulation by Ca^2+^-activated K^+^ (BK) channel to terminate Ca^2+^ influx for transmitter release. However, EJPs in *slo* NMJs are nearly normal. Therefore, it may be instructive to contrast the two types of synaptic homeostatic mechanisms, one induced by HT rearing and the other by hyperexcitability, to illustrate their distinct homeostatic adjustments to pre- and post-synaptic components (Figure 7).

Figure 7A illustrates the pre- and post-synaptic homeostasis mechanisms induced by *slo* BK channel mutations to highlight the corresponding physiological readjustments for achieving stability of synaptic transmission (Lee et al., 2008). The surprising finding of nearly normal EJP sizes in *slo* mutants reveals a compensatory upregulation of a 4-AP sensitive K^+^ current (likely Shaker I_A_) for the defective BK currents. In addition, a re-composition of postsynaptic transmitter receptor GluRII (ratio between A and B subunits) leads to reduced mEJP and mEJC sizes. Even though *slo* induces extensive overgrowth of functional satellite boutons, the combined effects of these two adjustments partially restore the EJP size (Figure 7A, Points 1, 2, and 3). It is important to note that one of the characteristic features of *rut* AC action is its dependence on activity-dependent Ca^2+^ influx (Dudai and Zvi, 1984; Livingstone et al., 1984), presynaptically through the *cac* Ca^2+^ channels and postsynaptically through *Dmca1D* Ca^2+^ channels (Lee et al., 2013). In *rut* mutants, neither pre- nor post-synaptic homeostatic adjustments occur (Lee et al., 2008).

In contrast, as shown in Figure 7B, the HT-rearing induced synaptic bouton overgrowth is counterbalanced by an increase in muscle membrane area. HT rearing does not change the properties of individual boutons, and hence does not cause alterations in mEJC size. However, mEJP size is decreased due to a decrease in muscle input resistance, presumably owing to an increase in membrane area. Although the increase in bouton numbers leads to more vesicle release (increased quantal content) and larger EJC size, it is counterbalanced by the decreased input resistance to produce a nearly normal sized EJP, the ultimate whole-cell functional parameter of neuromuscular transmission.

A number of issues still remain to be further investigated in terms of the down-stream targets of *rut* AC in these two cases of synaptic homeostasis. Several well-established molecular networks that function in the larval NMJ should facilitate the endeavor to work out the molecular framework for different cases of HT rearing- and hyperexcitability-induced synaptic homeostasis. It has been shown that cAMP-dependent protein kinase (PKA) mediates protein phosphorylation and plays a role in larval NMJ synaptic function, with mutations of PKA subunits partially reflecting *dnc* or *rut* defects (Renger et al., 2000). Furthermore, manipulations of cAMP response element binding protein CREB have been shown to control synaptic growth at larval NMJs (Davis et al., 1996; Schuster et al., 1996).

## Conflict of interest statement

The authors declare that the research was conducted in the absence of any commercial or financial relationships that could be construed as a potential conflict of interest.
